# Conceptualising the policy practice and behavioural research relationship

**DOI:** 10.1186/1479-5868-5-16

**Published:** 2008-03-20

**Authors:** Mark A Lawrence, Heather Yeatman

**Affiliations:** 1 School of Exercise and Nutrition Sciences, Deakin University, 221 Burwood Hwy, Burwood, VIC 3125, Australia; 2 School of Health Sciences, University of Wollongong, NSW 2522, Australia

## Abstract

**Background:**

Policy is frequently identified in the behavioural nutrition and physical activity research literature as a necessary component of effective research and practice. The purpose of this commentary is to promote a dialogue to contribute towards the further development of conceptual understandings and theories of the relationship between policy practice and behavioural research and how these two activities might work synergistically to improve public health outcomes.

**Methods:**

Drawing on policy and public health literature, this commentary presents a a conceptual model of the interaction and mediation between nutrition and physical activity-relevant policy and behavioural nutrition and physical activity research, environments, behaviours and public health implications. The selling of food in school canteens in several Australian states is discussed to illustrate components of the relationship and the interactions among its components.

**Results:**

The model depicts a relationship that is interdependent and cyclic. Policy contributes to the relationship through its role in shaping environmental and personal-cognitive determinants of behaviours and through these determinants it can induce behaviour change. Behavioural research describes behaviours, identifies determinants of behaviour change and therefore helps inform policy development and monitor and evaluate its impact.

**Conclusion:**

The model has implications for guiding behavioural research and policy practice priorities to promote public health outcomes. In particular, we propose that policy practice and behavioural research activities can be strengthened by applying to each other the theories from the scientific disciplines informing these respective activities. Behavioural science theories can be applied to help understand policy-making and assist with disseminating research into policy and practice. In turn, policy science theories can be applied to support the 'institutionalisation' of commitments to ongoing behavioural research.

## Background

Policy is recognised among behavioural researchers in nutrition [1] and physical activity [2-4] as integral to their research for promoting public health outcomes [5]. Despite its recognised importance, theoretical understanding of how to achieve the best fit between policy and behavioural nutrition and physical activity research is limited. As Ball and colleagues propose [6], progressing conceptual understanding and empirical evidence regarding the role of policy is among the "key priorities as part of a research agenda for advancing our understanding of environmental determinants of nutrition and physical activity behaviours".

For the purposes of this paper we define policy as, 'a statement of values, beliefs and intentions towards shaping the environmental (economic, social, physical and cultural) and personal-cognitive determinants that influence nutrition and physical activity behaviours'. The many definitions of policy within the policy science and public health literatures have created different interpretations of the role of policy in behavioural research. The definition used here allows exploration of the impact of policy on a range of environmental and personal-cognitive determinants of behaviour through legislative, education and social marketing instruments. This overarching role for policy is consistent with that outlined in the World Health Organisation framework for the implementation of the Global Strategy on Diet, Physical Activity and Health [7] in which 'Strategic policy and leadership' followed by 'Policy instruments' are the two preceding steps towards creating supportive environments for behaviour change.

Researchers and practitioners working either in the policy arena or in behavioural sciences need to develop stronger links in order to progress their agendas for mutual benefit. Whereas many practitioners are skilled at identifying problems and seeing what could be done, often they have less understanding of the role of policy and its capacity to create a supportive environment for behavioural initiatives to induce behaviour changes. Conversely, while there has been much progress in developing rules and procedures for evidence-based policy-making (and there is an expanding knowledge base regarding both nutrition and/or physical activity and health relationships) less is known about how evidence from behavioural research might best be translated into policy practice in the 'real world' [8].

If we are to promote public health, we need to build conceptual understandings and theories of the relationship between policy practice and behavioural research. The present paper aims to contribute to this agenda. Drawing on policy and public health literature, a conceptual model of the interaction and mediation between nutrition and physical activity-relevant policy and behavioural nutrition and physical activity research, environments, behaviours and public health implications is presented. The separate contributions of policy and behavioural research to public health outcomes are then examined. The model is illustrated through a case study of recent developments to sell healthier food in school canteens in New South Wales in Australia. Finally, the model's implications for priority research and policy practice agendas to promote public health outcomes are discussed.

## Discussion

### A conceptual model of the policy practice – behavioural research relationship

**Figure 1:**
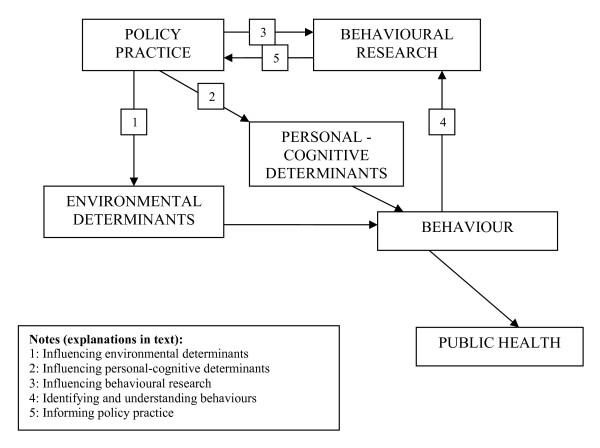
Conceptual model of the policy practice – behavioural research relationship.

A conceptual model of the interaction and mediation between nutrition and physical activity-relevant policy and behavioural nutrition and physical activity research, environments, behaviours and public health implications is depicted in figure 1. The model illustrates that the contributions of policy to behavioural research and behavioural research to policy practice are interdependent and cyclic, each complementing the other. Policy influences the environmental and personal-cognitive (including awareness, knowledge, attitudes and social norms, among many others) determinants of behaviours and via those determinants induces behaviour changes, for example a policy of mandatory nutrition information on food labels assists shoppers to select appropriate foods. In turn, such policy is dependent upon behavioural research identifying those determinants of behaviour that are amenable to policy directives, i.e. providing nutrition information at point of purchase facilitates healthy food choices. Behaviour changes are monitored and fed back iteratively to inform behavioural research, and subsequently policy practice.

This conceptual model consists of a number of components with complex interactions. The application of this model to professional practice requires practitioners to identify where their actions are located within the model. Appreciating how their specific activity interacts with other components of the model, will enable practitioners to undertake more strategic actions to promote public health outcomes. The specific contributions to public health outcomes of the policy practice and behavioural research components of the model are discussed in the following sections.

### The contributions of policy practice to public health outcomes

Policy practice can contribute to public health outcomes by influencing both the environmental and personal-cognitive determinants of behaviours, and via those determinants can induce behaviour changes, and the conduct of behavioural research.

#### Contribution 1: Influencing environmental determinants

Health status is not distributed equally among populations. Instead the social, economic, cultural and other circumstances in which people live play a significant role in health outcomes [9]. Policy has the ability to influence such environmental determinants by drawing upon a variety of instruments to mitigate disadvantageous circumstances and/or provide opportunities for health. This relationship has been understood broadly as one of helping 'make healthier choices easier choices' [10,11]. The effectiveness of information-based interventions in isolation from environmental considerations to influence behaviour and public health outcomes has been challenged [12,13]. When such interventions are complemented with policy commitments based on behavioural nutrition and physical activity research there can be an opportunity to help make choices not only more informed, but also more accessible and more available. This type of policy influence has been described by Milio [14] as using 'hard' policy instruments and they generally come at an economically and politically high cost. Examples include legal instruments, such as the indexing of welfare payments to the minimal cost of purchasing a diet consistent with the dietary guidelines, taxes on high fat foods and regulations to require bike paths as part of the built environment in local communities to help prevent obesity [15-17].

#### Contribution 2: Influencing personal-cognitive determinants

Policy can be used to influence personal-cognitive determinants of behaviours through instruments such as social marketing (promoting 2 fruit and 5 vegetables a day for a healthy diet) and education (curricula in schools). Social marketing often has been used by governments to target specific behaviours, placing the onus on individuals to enact recommended changes rather than attempting to bring about 'hard' political change. It can act to "make the recommended health behaviour more advantageous than the unhealthy behaviour it is designed to replace and then communicates the more favourable cost-benefit relationship to the target audience [18]." Such policy approaches are described by Milio [14] as 'soft' policy instruments. In placing the onus of responsibility onto the individual they generally carry less economic and political risk to the government.

#### Contribution 3: Influencing behavioural research

Policy can directly influence behavioural research itself through 'institutionalising' (securing ongoing commitments) a funding organisation's provision of resources for conducting research. For example, a government department may develop a policy that commits a specified fraction of its budget to behavioural research each year. A secondary influence of policy is the establishment of reference standards or procedures to inform policy objectives. For example a policy may mandate daily physical activity programs in primary schools. Behavioural research data are needed to help determine how much physical activity will produce the required effect or what type of activity is more appropriate and against which behaviour change can be measured.

### The contributions of behavioural research to public health outcomes

Behavioural nutrition and physical activity research contributes to public health outcomes by building the evidence base of behaviours, their determinants and their response to policy interventions, as well as helping inform policy-making practice.

#### Contribution 4: Identifying and understanding behaviours

Behavioural research can describe health behaviours across populations and help to identify and understand the environmental and personal-cognitive determinants of such behaviours. Moreover, behavioural research can monitor and evaluate behaviour change resulting from policy interventions, i.e. which policy instruments are effective in creating behaviour change, or what may be impeding the implementation of policies.

#### Contribution 5: Informing policy practice

Behavioural research can inform policy practice through disseminating to policy-makers research data on behaviours and the effectiveness, or otherwise, of previously implemented policy interventions. Policy practice is a human activity and therefore behavioural research also can inform the policy process itself by helping to identify what opportunities exist to develop policy and what influences decision-making.

#### Case study – Traffic light system in school canteens

**Figure 2:**
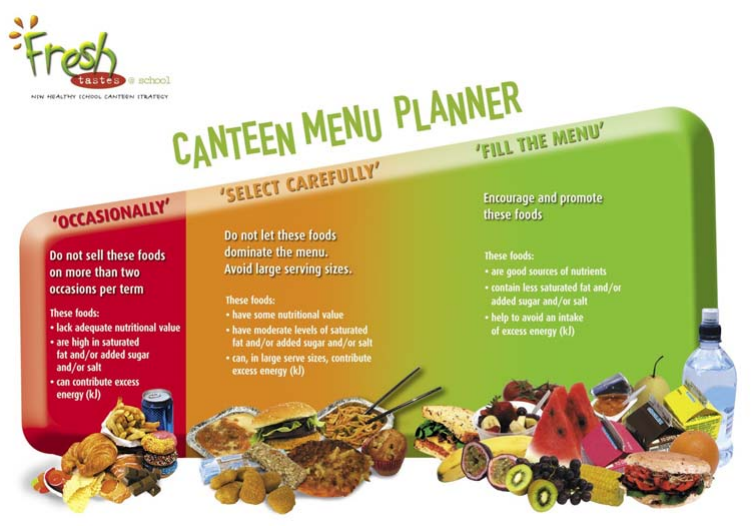
The traffic light system for planning canteen menus in NSW schools.

The implementation of a traffic light system to categorise foods in school canteens in several Australian states is a multifaceted policy that combines food availability and social marketing instruments. For instance, in New South Wales (NSW), the state government introduced a policy to control the foods bought and sold in school canteens. School lunch programs are not offered in Australian public schools. Students either bring their lunch from home, or purchase lunch from the school canteen. The policy is based around a traffic light system for planning canteen menus in NSW schools [19] and is represented in figure 2. Implementation of the policy is mandatory for public schools and voluntary for private schools. The 'Fresh Tastes Healthy Canteen Strategy' sits within the 'Healthier Schools' priority area of the NSW Government Action Plan for the Prevention of Obesity in Children and Young People 2003–2007 [20]. This approach requires schools to provide healthier food choices in line with the Australian dietary guidelines [21] and the Australian Guide to Healthy Eating [22].

The following components of the NSW state government's policy illustrate the interactions between the components of the conceptual model.

#### Policy practice contributions

##### i) Influencing environmental determinants

Policy mandates the types of foods available to children in the school canteen. It also shapes the environment for professional practice, as managers of school canteens have specific guidelines to follow in purchasing foods.

##### ii) Influencing personal-cognitive determinants

Policy requires the promotion of messages consistent with the national Dietary Guidelines and food selection guide. Teachers are encouraged to incorporate the canteen food selection guide and activities into lessons.

##### iii) Influencing behavioural research

Policy supports the provision of resources and other support for school-based research. The food categorisation system provides a reference standard against which school canteen practice and school children's behaviour can be monitored.

#### Behavioural research contributions

##### iv) Identifying and understanding behaviours

Behavioural research provides data to identify the determinants of behaviours within the school environment over which governments have control (and a duty of care) and hence can influence through policy. As well as providing feedback on the impact of policy – monitoring the impact of policy requires behaviour research techniques, for example monitoring food selection behaviours of children within and outside of school.

##### v) Informing policy practice

Pressures on schools to use canteens to raise school funds and existing attitudes of school staff relating to school canteens acted against the voluntary introduction of healthier foods. An analysis of decision-makers undertaken by behavioural researchers determined that behavioural interventions alone would be insufficient and legislation was required as a core component during the policy-making process.

Following the introduction of the traffic light system, evaluation has identified that the vast majority of school canteen managers report understanding the menu planning tool and making changes, such as limiting the sale of all red foods. School-wide changes have been reported, including the establishment of canteen committees, development of school canteen policies, involvement of the school community and promotion of healthier products. Importantly, school canteen managers reported changes in their attitudes regarding the role of school canteens in supporting the curriculum and improving the nutritional health of children and young people, and only 5% reported a loss of profits from the canteen [23].

### Implications for research and policy practice

The conceptual model's practical value is its role in guiding behavioural research and policy practice activities to promote public health outcomes. In this section we describe a priority research agenda for the different pathways within the model and discuss evidence-based recommendations for policy practice based on the model.

#### 1. Research priorities

##### i) Identifying and understanding behaviours

The operation of the model is dependent upon the ongoing availability of high quality evidence derived from nutrition and physical activity behaviour research. For example, the New South Wales Childhood Obesity Summit in 2002 was able to draw on evidence of a relationship between the ready availability of relatively cheap high fat, salt and sugar-containing foods, poor dietary behaviours and a high rate of childhood obesity to recommend a new school canteen policy. These data, together with existing Australian dietary guidelines for children [21] and school canteen actions [24] lead to the mandatory 'traffic light' food program for school canteens, referred to earlier in this paper. From a policy perspective it is critical that behavioural research be linked with strengthening understandings of the environmental and personal-cognitive determinants that influence behaviours. A priority research agenda will be to identify and understand those determinants that can be targeted by policies to help tackle the persistent social inequities in behaviour profiles and health outcomes among populations.

In the future, policy and behavioural scientists will continue to benefit if they collaborate to take advantage of opportunities to monitor and evaluate policy interventions. Such monitoring and evaluation activities will provide feedback into further policy development, in a similar way to that proposed in Rothman's work on theories of behaviour change, defining them as dynamic entities that need to be applied, tested and refined through interventions [25]. By working in partnership in the design of evaluation approaches, policy and behavioural scientists will be especially effective in determining whether a policy has actually been logically theorised, planned and resourced, i.e. framing its 'evaluability assessment' [26].

There is much debate about what evidence should be applied to public health policy-making [27,28]. Central to the debate is the nature and definition of evidence and how the quality of evidence and effectiveness is defined. Evidence-based medicine has developed a rigid hierarchy of rules for assessing the quality of scientific evidence that relates to the nature of study design [29]. This system has proved effective in enabling comparisons of the relative merit of single clinical interventions. However, in the future a research priority will be to adapt conventional rules and procedures of evidence-based medicine to a public health perspective. The complex, interactive and social nature of public health policy interventions make them significantly different from physiological or clinical interventions where there is a higher degree of control and little influence from social factors [30,31]. For example, if we are to account for the determinants of behaviours, policy-making in public health needs to include all information of relevance, including the organisational and social context in which it was gathered [32]. Daly et al have made an important contribution to the area by proposing a hierarchy for assessing evidence obtained from qualitative methods that serves as a guide for the critical appraisal of papers using qualitative methods and provides a basis for informing policy-making [33].

##### ii) Informing policy practice

The principle of public health policy decisions being informed by scientific evidence, with its implicit notion of rationality and freedom from ideology, has an inherent appeal [34]. Certainly, the degree of behavioural research activity coupled with the investment in research methods and systematic review procedures have provided policy-makers with an evidence base of unprecedented quality and quantity. Yet, the availability of evidence is not necessarily the 'rate-limiting step' in policy-making. Many scientists bemoan that their evidence is not used by policy-makers and many policy-makers claim that scientists are not supplying policy relevant evidence [35,36]. Policy-makers need support to utilise evidence and researchers need to become more cognisant of policy requirements. As Hanney et al argue the failure of the so-called 'two communities' to communicate has resulted in researchers pursuing their own agendas, rather than those of the potential users of the research [37].

Clearly, more needs to be known about how evidence is translated into policy and, conversely, how policy requirements might receive more attention within research activities. A priority research agenda is the relatively new field of inquiry of knowledge translation and exchange to assist with the dissemination of research findings into policy and practice [38]. Increasingly, critical roles in helping understand the evidence-policy linkages and promoting knowledge translation and exchange are being undertaken by expert agencies [39-41] dedicated to developing partnership arrangements between researchers and research users throughout the research process [42]. For example, the Research Unit for Research Utilisation in Scotland has developed a taxonomy of interventions to serve as a theoretical and practical tool for conceptualising the research impact field and to help enhance the impact of research on policy and practice [43]. Also, it has prepared systematic reviews of the 'diffusion of innovation' and the 'knowledge management' literature [44,45], providing useful building blocks for further unpacking the dynamics of evidence use in day-to-day service delivery.

Improved two-way communication between researchers and policy-makers may improve the uptake of research evidence [46] but evidence, even when translated and acknowledged, is not of itself sufficient to bring about policy change. Behavioural scientists understand that people will not necessarily change their nutrition or physical activity actions based on information alone – why are they surprised that policy-makers act in the same way? Policy is, by definition, a political action, subject to a range of human, social and economic influences. Irrespective of how compelling the evidence for a problem may be, public health policies do not arise of their own volition. Instead, the values, beliefs and interests of stakeholders [47-49], the coalitions formed between like-minded stakeholders [50], and the political circumstances [51] have all been identified as critical in explaining evidence-based practice in policy-making.

Therefore, other types of theoretical understandings, research designs, methods and measures are required if we are to be effective in acting on evidence to promote public health outcomes. Policy science research involving the critical analysis of case studies of policy-making processes can provide valuable insights into the interrelationships between the components of the model so they can be used to predict and explain policy change [52]. For example, in NSW a case study research design has been used in analyzing health impact assessment informing government health policy [53]. The findings of such case studies highlight that the mechanisms for linking research with policy and practice extend beyond communication channels between individual researchers and government officials. Instead, as Bowen and Zwi argue there exists a variety of individual, organisational, and system-level values that influence how evidence is sourced, used and implemented in policy-making [54]. They propose an "evidence-informed policy and practice pathway" to help explain the use of evidence" in policy-making.

Policy-makers require information to be framed to achieve particular outcomes and to reinforce their current understanding of an issue or to shift them from their current position. In other words, information is required to meet their particular stage of policy change. As Armstrong et al [55] conclude from their analysis into the behaviours of public health policy-makers, the translation of knowledge into policy practice requires knowledge management processes and practitioner engagement strategies to complement the scientific evidence on offer. Glasgow and Emmons also highlight the importance of contextualising (what are the circumstances of a relationship?) and explaining the external validity (the generalisability to various persons, settings, etc) of evidence, to assist decision-makers in their policy-making activities [56].

Ultimately, creating political will and subsequently making policy is about influencing attitudes and behaviours [57]. Insights from behavioural science need to be adapted and extended to the special case of behaviours of policy makers. Behavioural scientists need to undertake more strategic policy actions to achieve public health outcomes [58]. It is in this context that behavioural science theories provide especially relevant understandings for research into the power relations and the psychology of decision-making processes in policy-making. For example, how the policy agenda is framed via the defining of risks and benefits, giving legitimacy to certain measures, the provision of resources and the framing of rules and procedures for who, how, and when data are collected and analysed.

#### 2. Policy practice priorities

##### i) Influencing environmental and personal-cognitive determinants

Traditionally, policy practice to promote public health outcomes has tended to be initiated by the health sector within government and targeted at individual behaviours [59]. Such policy practice generally has failed to engage those sectors that have the greatest influence over the environmental determinants of health behaviour. Our understanding of effective policy practice for influencing environments is constantly improving. Analytical instruments for targeting policies to create environmental change have been proposed [60]. Such instruments recognise that health behaviours result from a complex mix of underlying factors. Hence, policy practice needs to adopt a systematic and comprehensive approach if it is to address the many underlying factors associated with health behaviours.

The importance of policy approaches that address the multiple levels of government, i.e. local, state and/or regional, national and international and the intersectoral nature of the determinants of behaviours has been emphasised [59]. A review of such policy approaches has been provided by Sacks et al to provide evidence-based recommendations for undertaking systematic policy practice for obesity prevention [15]. These recommendations are based on the concept of a grid system to policy practice in which the levels of governance and the relevant sectors form the two dimensions of the grids for planning physical activity and nutrition policy interventions.

##### ii) Influencing behavioural research

In the future, the priority agenda for policy practice to influence behavioural research will be two-fold. Firstly, to put in place policies that will help secure commitments for the ongoing conduct of behavioural research. As with policy interventions in general, this activity will involve policy practitioners and research scientists combining to advocate for such policies and identify strategically where and when such advocacy should be undertaken. A second priority agenda will be to establish, if not already in existence, and update reference standards for nutrition and physical activity against which behavioural research can be framed for assessing policy performance.

## Conclusion

The relationship between policy practice and behavioural nutrition and physical activity research is complex and our understanding of its pathways and operation is in its early stages of development. The commentary we have presented here provides a contribution to the conceptual and theoretical understanding of the relationship.

We assert that the relationship is cyclic and its components are interdependent. There is a need for behavioural scientists to provide evidence to stimulate policy initiatives, to apply their theories to policy-making practice and to monitor and evaluate policy change. Equally, there is a need for policy practitioners to develop policies that build commitments to ongoing behavioural research and tackle the determinants of behaviours identified by behavioural scientists. Hence, strengthening the relationships between these two sets of activities is mutually beneficial.

Attempting to promote behaviour research or policy practice in isolation of the other dimension is akin to tackling the public health challenges and obstacles with one hand tied behind our backs. In the future we look forward to behavioural researchers and policy practitioners collaborating in even stronger partnerships to pursue common goals and activities in promoting nutrition and physical activity behaviours and ultimately public health.

## Competing interests

The author(s) declare that they have no competing interests.

## Authors' contributions

ML conceived and initiated the original manuscript. Both authors drafted the manuscript. Both authors read and approved the final manuscript.
